# Pathological effects of ionizing radiation: endothelial activation and dysfunction

**DOI:** 10.1007/s00018-018-2956-z

**Published:** 2018-10-30

**Authors:** Bjorn Baselet, Pierre Sonveaux, Sarah Baatout, An Aerts

**Affiliations:** 1 Radiobiology Unit, Belgian Nuclear Research Centre (SCK•CEN), Mol, Belgium; 2 Institute of Experimental and Clinical Research (IREC), Pole of Pharmacology and Therapeutics, Université catholique de Louvain (UCL), Brussels, Belgium; 3 Department of Molecular Biotechnology, Ghent University, Ghent, Belgium

**Keywords:** Vascular tone, Procoagulation, Prothrombosis, Endothelial cell retraction, Mitochondrial dysfunction, Premature senescence

## Abstract

The endothelium, a tissue that forms a single layer of cells lining various organs and cavities of the body, especially the heart and blood as well as lymphatic vessels, plays a complex role in vascular biology. It contributes to key aspects of vascular homeostasis and is also involved in pathophysiological processes, such as thrombosis, inflammation, and hypertension. Epidemiological data show that high doses of ionizing radiation lead to cardiovascular disease over time. The aim of this review is to summarize the current knowledge on endothelial cell activation and dysfunction after ionizing radiation exposure as a central feature preceding the development of cardiovascular diseases.

## Introduction

For many years after its discovery in the 1800s, the vascular endothelium was believed to be a mere inert, semipermeable barrier between circulating blood and underlying subendothelial tissues. Numerous subsequent studies have led to the current view of the endothelium as a dynamic heterogeneous and distributed organ with essential secretory, synthetic, metabolic, and immunologic functions [1]. In the presence of irritant stimuli, such as dyslipidemia [2, 3], hypertension [4–7], and pro-inflammatory agents [8–11], the normal physiological functions of the arterial endothelium are adversely affected [12, 13], starting a chain of molecular changes that leads to atherosclerosis and cardiovascular diseases (CVDs), including coronary artery disease, carotid artery disease, peripheral artery disease, and ischemic stroke [14–16].

When cells are exposed to ionizing radiation, they undergo a stress response within less than a microsecond after the hit [17]. This response is initiated by the interaction of ionizing radiation with biological matter, causing damage by interacting directly or indirectly through the formation of reactive oxygen species (ROS) with cellular biomolecules such as DNA, proteins, and lipids. This reaction interferes with all cellular organelles and has the ability to affect their molecular mechanisms. As a result, endothelial activation occurs, causing the quiescent phenotype to switch towards a pro-inflammatory one [18–20]. When exposure is prolonged and/or repeated, it can exhaust the protective physiological effect of the endothelium, leading to endothelial dysfunction [21]. This pathological state can thus be seen as a maladaptive response to pathological stimuli and refers to a failure of the endothelium to perform its normal, physiologic functions [22]. As a result, deterioration of the vascular tone, blood hemostasis problems, inflammation, and edema occurs at the site of the affected endothelium [23]. Because the endothelium is a key integrator of vascular risk, pathogenic signals, including ionizing radiation, may converge to produce several pathological conditions [22], atherosclerosis as typified example [24]. Atherosclerosis itself perpetrates vascular damage, resulting in radiation-induced heart disease [25, 26]. According to the current consensus, the term “low dose” is defined as a dose of 0.1 Gy or less throughout this review [27, 28]. The terms “moderate dose” and “high dose” are, respectively, defined in this review as doses between 0.1 Gy and 2 Gy, and equal or higher than 2 Gy. It has been shown that high doses of ionizing radiation-induced cardiovascular diseases in atomic bomb survivors [29] and cancer therapy patients [30].

In the sections below, we summarize current knowledge on the effects of ionizing radiation exposure on the different aspects of endothelial activation and dysfunction. Progress in the knowledge of endothelial pathophysiology has mainly been a consequence of investigations performed with endothelial cells in culture (Table 1) [31], human umbilical vein endothelial cells (HUVECs) being the most commonly used [32]. To standardize experimental conditions, immortalized, well-characterized endothelial cell lines were developed, of which EA.hy926 is the most frequently used [33]. Additional models of endothelial pathophysiology encompass in vitro co-culture and 3D models that mimic in vivo complexity [34–38]. Ex vivo explanted blood vessel segments from animal (commonly used are canine, bovine, porcine, rat and mouse) or human origin are also used, mainly in the field of vascular tone research [39]. Finally, a number of animal models have been used for understanding the mechanisms involved in cardiovascular disease development as they can replicate complex cell–cell and cell–matrix interactions. The most common animal model being the ApoE^−/−^ mice [40, 41]. However, it is difficult to draw general conclusions, because the current literature often describes different endothelial cell models, timepoints, and radiation doses.

**Table 1:** Non-exhaustive list of the most commonly used endothelial cell models in endothelial pathophysiological research

**Primary cells**
Human umbilical vein endothelial cells (HUVEC)
Human aortic endothelial cells (HAEC)
Human coronary artery endothelial cells (HCAEC)
Human dermal microvascular endothelial cells (HDM(V)EC)
Human brain microvascular endothelial cells (HBM(V)EC)
Human ovarian microvascular endothelial cells (HOM(V)EC)
Human pulmonary microvascular endothelial cells (HPM(V)EC)
Human pulmonary aortic endothelial cells (HPAEC)
Human hepatic sinusoidal endothelial cells (HHSEC)
Human iliac vein endothelial cells (HIVEC)
Human placental endothelial cells (HPEC)
Bovine aortic endothelial cells (BAEC)
Bovine pulmonary artery endothelial cells (BPAEC)
Bovine adrenal microvascular endothelial cells (BAM(V)EC)
Mouse aortic endothelial cells (MAEC)
Mouse pulmonary microvascular endothelial cells (MPMEC)
Mouse cardiac microvascular endothelial cells (MCM(V)EC)
Rat aortic endothelial cells (RAOEC)
Rabbit aortic endothelial cells (RAEC)
**Cell lines**
EA.hy926 (HUVEC—human lung carcinoma cell line A549 hybridoma)
SV40-immortalized human dermal microvascular endothelial cells (HMEC-1)
Telomerase-immortalized human microvascular endothelial cells (TIME)
Telomerase-immortalized human coronary artery endothelial cells (TICAE)
SV40-immortalized human aortic endothelial cells
bEnd.3 (mouse brain microvascular endothelial cell line)
mIEnd1 (mouse endothelioma cells)
**2D co-cultures**
Endothelial cells—fibroblasts
Endothelial cells—smooth muscle cells
Endothelial cells—fibroblasts—smooth muscle cells
**3D models**
**Ex vivo explants**
Human umbilical cord rings
Human cervical artery
Human axillary artery
Rabbit abdominal/thoracic aorta
Rabbit central ear artery
Rabbit carotid artery
Rat abdominal/thoracic aorta
**In vivo models**
Mouse
Rat
Rabbit
Pig
Dog
Non-human primates

*SV40* simian vacuolating virus 40

## Endothelial activation: a pro-inflammatory state

**Fig. 1:**
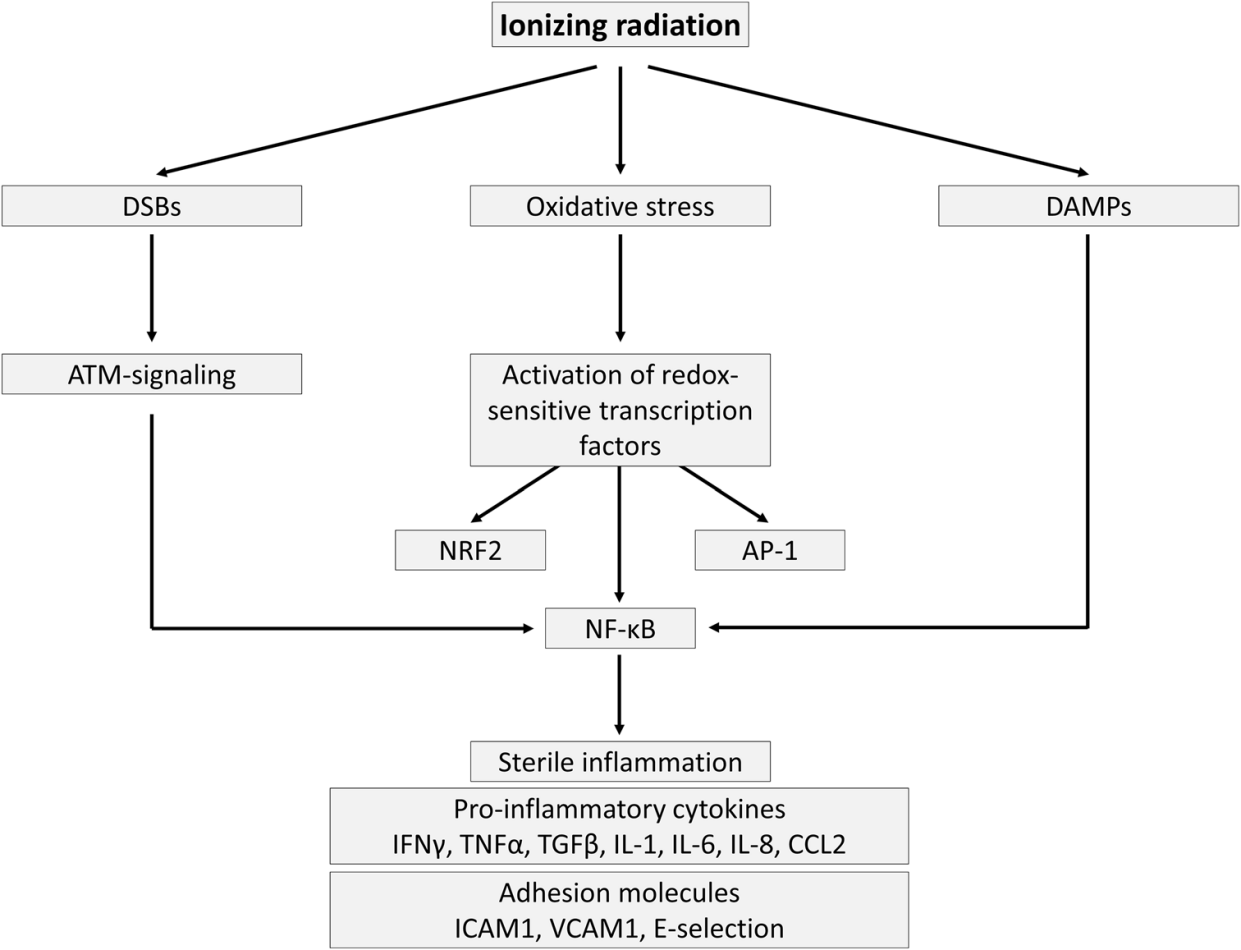
Radiation-induced sterile inflammation in endothelial cells. Ionizing radiation exposure activates redox-sensitive transcription factor NF-κB via DSB and ATM signaling, induces oxidative stress, and triggers the release of DAMPs. The resulting inflammation leads to the production and secretion of pro-inflammatory cytokines as well as to the expression of a modified repertoire of adhesion molecules by irradiated endothelial cells

Endothelial cell activation can be defined by the manifestation of a pro-inflammatory phenotype characterized by the expression of chemokines, cytokines, and adhesion molecules that facilitate the recruitment and attachment of circulating leukocytes on the vascular wall [21]. Endothelial cells are typically activated by pro-inflammatory cytokines tumor necrosis factor (TNF)-α and interleukin (IL)-6, released by immune cells upon contact with pathogens [42]. After ionizing radiation exposure, however, endothelial cell activation occurs in a sterile environment without the presence of pathogens, i.e., sterile inflammation (Fig. 1). The prime cause of sterile inflammation following ionizing radiation exposure is activation of the genotoxic stress-induced nuclear factor (NF)-κB pathway, recently reviewed by Hellweg [43]. NF-κB is a heterodimeric transcription factor that is normally sequestered in the cytoplasm as an inactive complex with inhibitor of κB (IκB) [44]. DNA double-strand breaks (DSBs), produced by direct or indirect radiation damage to DNA, act as an initial trigger that results in activation of ataxia telangiectasia mutated protein (ATM). Activated ATM promotes nuclear export of IKK-γ/NF-κB essential modulator (NEMO), a regulatory subunit of the IκB kinase complex that is able to activate NF-κB in the cytoplasm. During this process, a nucleoplasmic signalosome is required for NEMO posttranslational modification and NEMO shuttling to the cytoplasm. While the composition of the nucleoplasmic signalosome is not fully elucidated, p53-induced protein with a death domain (PIDD), receptor interacting protein 1 (RIP1), and poly(ADP-ribose)-polymerase-1 (PARP-1) are known to play supporting roles [43]. In this context, a dose of 8–10 Gy of either γ-rays or X-rays was found to activate the genotoxic stress-induced NF-κB pathway in HUVECs [45, 46].

Another possible cause of sterile inflammation is oxidative stress, a recognized consequence of endothelial cell exposure to radiation (Fig. 1) [47–50]. Besides reacting with cellular biomolecules, ROS directly activate redox-sensitive transcription factors nuclear factor (erythroid-derived 2)-like 2 (NRF2), activator protein 1 (AP-1), and NF-κB [44]. AP-1 is a heterodimeric transcription factor composed of members of the Jun, Jun dimerization protein (JDP), FOS, and related activating transcription partner families [51, 52]. Depending on its composition, it plays a role in the expression of several genes involved in cellular differentiation, proliferation, and apoptosis. Examples of AP-1-target genes are transforming growth factor (TGF)α, TGFβ, and IL-2 [51]. Activation of AP-1 during oxidative and inflammatory stimuli is predominantly mediated by mitogen-activated protein kinase (MAPK) signaling [44]. NF-κB is also a redox-regulated transcription factor: inflammatory and/or oxidative stimuli activate a series of upstream kinases, such as MAPKs, IκB kinase, protein kinase C (PKC), and phosphatidylinositide 3-kinases (PI3 K), which then activate NF-κB by phosphorylation-mediated degradation of IκB. Activated NF-κB translocates to the nucleus and induces the expression of a wide array of genes regulating pro-inflammatory mediators TNF-α, IL-8, IL-1, inducible nitric oxide synthase (iNOS), and cyclooxygenase-2 [44]. In endothelial cells, NF-κB is involved in the transcriptional regulation of most cytokines and adhesion molecules [53–57].

Another possible cause of endothelial activation is the release of damage-associated molecular patterns (DAMPs) by stressed and dying cells (Fig. 1). Tissue injury emits DAMPs that serve as danger signals to activate danger control (i.e., inflammation for host defense). DAMPs can either be intracellular molecules that signal cell stress and necrosis [high-mobility group box 1 (HMGB1), histones, purine metabolites, uric acid, S100 proteins, heat-shock proteins, and DNA/RNA outside nucleus or mitochondria], matrix constituents that signal extensive matrix remodeling (hyaluronan fragments and glycosaminoglycan fragments) and luminal factors that signal barrier destruction (uromodulin, oxidized low-density lipoprotein). DAMPs activate toll-like receptors, purinergic receptors, and inflammasomes in parenchymal cells and leukocytes. DAMP binding on endothelial cells upregulates pro-inflammatory signaling pathways that lead to NF-κB, MAPK, and interferon regulatory factor 3 (IRF3) signaling [58, 59], resulting in expression of adhesion molecules [intercellular adhesion molecule (ICAM)-1 and vascular cell adhesion molecule (VCAM)-1, and E-selectin] and the release of cytokines [IL-6, IL-8, chemokine C–C motif ligand (CCL) 2, and interferon (IFN) γ] [42, 60–63]. In this respect, exposure to doses ≥ 2 Gy of X-rays was found to induce a dose-dependent in vitro and in vivo release of HMGB1 [64], known to induce endothelial expression of IL-6, CCL2, ICAM-1, and VCAM-1 [65]. In the murine microvascular endothelial cell line, bEnd.3, irradiation with 10 Gy has been shown to promote *HMGB1*gene expression [66]. Moreover, NF-κB signaling was found to be upregulated in irradiated arteries of patients treated with radiotherapy, even months or years after radiation exposure [67].

In general, high doses (> 2 Gy) of ionizing radiation induce endothelial activation. Endothelial adhesion molecules ICAM-1 and E-selectin are upregulated in a time- and dose-dependent manner [68–70], in part due to NF-κβ activation [71]. Furthermore, the expression of cytokines IL-6 and IL-8 as well as TGF-β was shown to increase after exposure to high doses of ionizing radiation [18, 72, 73] and was further differentially affected by dose quality [74]. For example, in obese ApoE^−/−^ mice, a 14 Gy exposure induced an inflammatory phenotype, accelerating atherosclerotic plaque formation and rupture [75]. In addition, atomic bomb survivors exposed to high doses are more prone to the development of atherosclerosis [29] and demonstrated signs of general inflammation, with increased levels of IL-6 and C-reactive protein (CRP) [76]. Comparatively, the effects of low doses (≤ 2 Gy) of ionizing radiation on endothelial activation are still under debate (also discussed in [77]). On one hand, increased ICAM-1 expression and concomitant leukocyte attachment were detected in in vitro endothelial cell cultures after 0.125–0.5 Gy [78]. In addition, we detected elevated IL-6 and CCL2 levels in human endothelial cells exposed to 0.5 Gy [79]. On the other hand, a decrease in endothelial ICAM-1 and E-selectin expression has been observed after exposure of mice to 0.3 Gy and 1 Gy [69], which caused decreased endothelial adhesiveness to monocytes [69, 80]. This anti-inflammatory effect of low-dose radiation, which was confirmed by others [50, 81–86], requires a pre-activation of endothelial cells with pro-inflammatory stimuli TNF-α, IL-1β, or lipopolysaccharide. When these mice were exposed to low amounts of ^137^Cs delivered in the drinking water, the pro-inflammatory plaque phenotype was diminished [87]. The dampening effect of radiation exposure on endothelial activation has been used for decades for the treatment of benign inflammatory diseases [88, 89]. Today, the use of low-dose radiotherapy for the treatment of chronic inflammatory diseases is rare, due to the debate on possible cancer and non-cancer risks [85].

It must be emphasized that endothelial cell activation is a normal part of bodily defense mechanisms. In physiological circumstances, it draws immune cells to sites of infection or tissue injury. The difference between normal physiological and detrimental pathological activation of the endothelium lies in the nature, extent, duration, and combination of pro-inflammatory stimuli. As a consequence of prolonged and/or repeated exposure to a combination of cardiovascular risk factors, the protective effect of endogenous anti-inflammatory systems of endothelial cells can ultimately be depleted, resulting in endothelial dysfunction [21]. An overview of findings supporting endothelial inflammation in different endothelial cell cultures and organs by different radiation qualities and doses is given in Table 2.

**Table 2:** Experimental findings to support the induction of an endothelial pro-inflammatory state by ionizing radiation

Time factor	Experimental model	Radiation quality (dose rate)	Total dose (Gray)	Experimental findings	Methods	References
Acute	HUVEC	γ-rays (Cs-137, 2 Gy/min)	8	DNA-binding activity of NF-κB 6 h after irradiation	EMSA	[45]
Acute	HUVEC	X-rays	10	DNA-binding activity of NF-κB at 30–60 min after irradiation	EMSA	[46]
Acute	HUVEC	X-rays	0.5, 10, 20	Induced E-selectin expression 4 h after irradiation	Flow cytometry and northern blot analysis for E-selectin	[46]
Acute	bEnd.3	X-rays	10	HMGB1 gene expression increased 24 h after irradiation	RT-qPCR	[66]
Fractionated	Human cervical artery	X-rays	50–68	NF-κB activation in irradiated human arteries 4–500 weeks after radiotherapy	Gene expression profiling, immunofluorescence for NF-κB p65, CD68, CD3, MMP-1	[67]
Acute	HMVEC	X-rays	2, 5, 10, 20	Induced ICAM-1 expression 24–72 h after irradiation	Immunofluorescence for ICAM-1	[70]
Acute	EA.hy926	X-rays (0.813 Gy/min)	0.3, 1, 5	Induced E-selectin expression 1–5 h after irradiation	Flow cytometry and enzyme-linked immunosorbent assay (ELISA) for E-selectin	[69]
Acute	HUVEC	X-rays	7	NF-κB induced ICAM-1 and E-selectin gene expression 6 h after irradiation	Flow cytometry and promoter–reporter construct transfection for E-selectin and ICAM-1	[71]
Acute	HUVEC	γ-rays (Co-60, 1 Gy/min)	10	Elevated IL-6, IL-8 and IL-10 production 3 days after irradiation	ELISA for IL-6, IL-8 and IL-10	[72]
Acute	HMVEC	γ-rays (Cs-137, 1 Gy/min)	10	Elevated active and total TGFβ1 production 24 h after irradiation	ELISA for TGF-β1	[73]
Acute	TICAE	X-rays (1.5 Gy/min) Fe ions (1.5 Gy/min)	2	Elevated IL-6 and IL-8 production after X-ray exposure and not after iron irradiation	ELISA for IL-6 and IL-8	[74]
Acute	TICAE	X-rays (0.5 Gy/min)	0.5, 2	Elevated CCL2 and IL-6 production 1–7 days after irradiation	Multiplex bead array	[18]
Acute	ApoE^−/−^ mice	X-rays	14	Elevated number of hemorrhage-prone inflammatory atherosclerotic lesions	Hematoxylin and eosin staining, immunohistochemical staining for Mac3	[75]
Acute/fractionated (2 sessions)	HUVEC	X-rays (0.094 mGy/min)	0.125, 0.25, 0.5	Elevated NF-kB activation and ICAM-1 protein expression 18 h after both exposure types; greater ICAM-1 response after dose fractionation	Surface enzyme immunoassay for ICAM-1; ELISA for phospho-NF-kB p65 protein	[78]
Acute/fractionated (3 sessions)	mlEnd1	X-rays (1.15 Gy/min)	0.1–0.5	Reduced PBMC binding to endothelial cells 4–24 h after irradiation	PBMC adhesion assay after IL-1β-induced endothelial activation	[80]
Acute	EA.hy926	X-rays (4 Gy/min)	0.5	Reduced PBMC binding to endothelial cells 24 h and 48 after irradiation	PBMC adhesion assay after TNF-α-induced endothelial activation	[81, 82]
Acute/fractionated (2 sessions)	EA.hy926	X-rays (1.15 Gy/min)	0.5	Decreased CCL20 production by nonactivated endothelial cell and PMN co-culture; PMN binding to endothelial cell 24 or 48 h after, respectively, acute or fractionated irradiation	ELISA for CCL20; PMN adhesion assay after TNF-α-induced endothelial activation	[83]
Acute	Unspecified	Unspecified	0.7	Reduced PBMC binding to endothelial cells 4 h after irradiation	PBMC adhesion assay after IL-1β-induced endothelial activation	[84]
Acute	EA.hy926	X-rays (1.15 Gy/min)	0.5	Reduced PBMC binding to endothelial cells 4 and 24 h after irradiation; DNA-binding activity of NF-κB maximal both 4–8 and 24–30 h after irradiation	PBMC adhesion assay after TNF-α-induced endothelial activation; EMSA	[86]
Chronic	ApoE^−/−^ mice	γ-rays (Cs-137)	20 or 100 kBq/l per day	Reduced gene expression of pro-inflammatory factors (CRP, TNF-α, CCL2, IFNγ), adhesion molecules (ICAM-1, VCAM-1, E-selectin) and reduced macrophage content in atherosclerotic plaques 6–9 months chronic radiation exposure	RT-qPCR for CRP, TNF-α, CCL2, IFNγ, ICAM-1, VCAM-1, E-selectin; Immunofluorescence for CD68	[87]

*Co* Cobalt, *Cs* Cesium, *EMSA* electrophoretic mobility shift assay, *Mac3* macrophage marker 3, *MMP*-*1* matrix metalloproteinase 1, *PBMC* peripheral blood mononuclear cells, *PMN* polymorphonuclear leukocytes, *RT*-*qPCR* reverse transcriptase real-time quantitative polymerase chain reaction

## Deterioration of the vascular tone

**Fig. 2:**
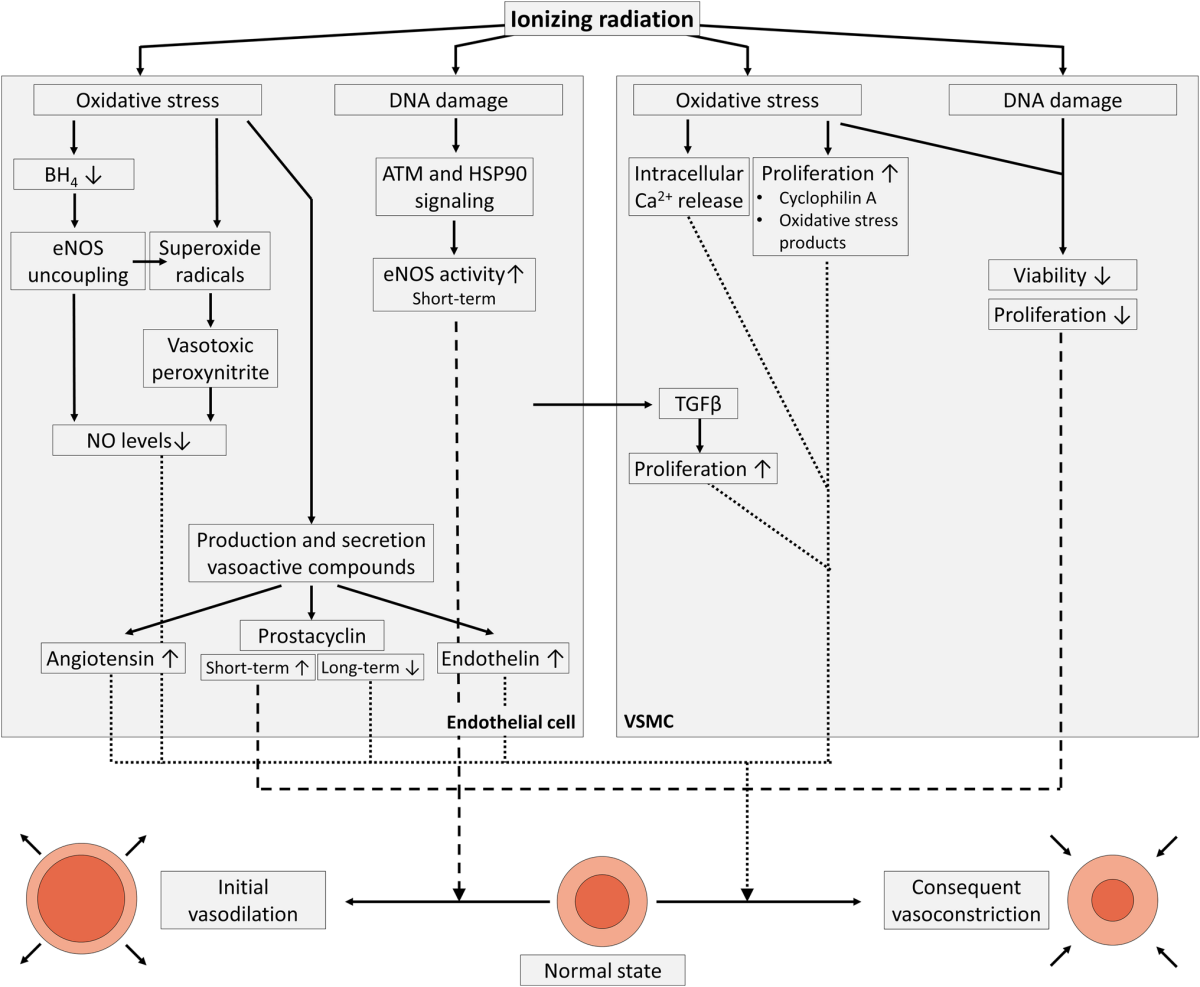
Irradiation-induced deterioration of the vascular tone. Ionizing radiation exposure induces oxidative stress and DNA damage in endothelial cells (left), leading to decreased NO levels and altered production and/or secretion of vasoactive compounds resulting in an initial vasodilation followed by vasoconstriction. In addition, VSMC irradiation induces oxidative stress and DNA damage, resulting in an initial reduction of cellular viability and proliferation as well as vasodilation (right). In the long run, oxidative stress results in Ca^2+^ release from intracellular stores and increased VSMC proliferation, supporting vasoconstriction

One of the key consequences of endothelial dysfunction is impairment of endothelium-dependent vasodilation due to reduced bioavailability of vasodilators, particularly nitric oxide (NO), and/or to elevated levels of endothelium-derived contracting factors, i.e., endothelins, prostaglandin, and thromboxane [23, 90–93]. The role of NO and its reactive intermediates in the endothelial radiation response largely remains an open question [94]. What is known is that, after exposure of endothelial cells to ionizing radiation, NO is rapidly deactivated by superoxide radicals, resulting in the formation of vasotoxic peroxynitrites [95, 96] (Fig. 2). Irradiation-induced oxidative stress also causes endothelial NO synthase (eNOS) uncoupling due to inadequate availability of its redox-sensitive cofactor tetrahydrobiopterin, resulting in eNOS-dependent production of superoxide and diminished release of NO [97]. From 1 to 4 days after irradiation, doses of 6 Gy and higher were found to promote eNOS expression and activity, leading to NO production and NO-induced angiogenesis with a concomitant increase in tumor blood flow [98, 99]. eNOS activation after endothelial irradiation depends on components of the DNA damage response pathway, namely, ATM and heat-shock protein 90, which phosphorylate Ser1179 of eNOS, leading to enhanced eNOS activity [100]. However, most of endothelial DNA damage signaling ceases within 24 h after irradiation [79], explaining why irradiation acutely but not chronically enhances NO availability. At later timepoints, endothelium-dependent vasodilation is compromised. Timing also depends on the dose and on the nature of the irradiated endothelial bed. For example, reduced endothelium-dependent vasodilation was found in rabbit carotid arterial rings 20 h after irradiation with 8 Gy and 16 Gy [95], in rabbit ear arteries 1 week after irradiation with 10 Gy, 20 Gy, and 45 Gy [101, 102], in rabbit aorta 9 days after whole-body irradiation with 1 Gy, 2 Gy, and 4 Gy [103] and in rat aorta 6 months after irradiation with 15 Gy [104]. In humans, endothelium-dependent vasodilation was found to be impaired both in vitro and in vivo in carotid arteries 4–6 weeks after neck irradiation (total pre-operative dose of radiation averaged 47.9 Gy ± 2.8 Gy) [105]. In addition, impaired endothelium-dependent vasodilation of axillary arteries was reported in breast cancer radiotherapy patients more than 3 year after radiotherapy (no average dose assigned) [106].

NO is not the sole vasoactive substance produced and released by the endothelium. The production of prostacyclin, a potent endothelium-derived vasodilator, is also affected by radiation exposure. Basal prostacyclin release was found to be unaffected in irradiated HUVECs at doses up to 25 Gy [107]. However, when endothelial cells were stimulated with exogenous arachidonic acid, a precursor of endothelial prostacyclin, prostacyclin levels decreased 15 min after irradiation [108], increased within 1 day after irradiation [109–113] and then decreased again thereafter in a radiation dose-dependent way [107, 111, 114, 115]. The short-term stimulatory effect of radiation on prostacyclin production is believed to be caused by oxidative stress [116, 117] and cell damage [111]. Endothelium-dependent hyperpolarization-related signaling was unaffected after endothelial irradiation, thereby serving as a reserve defense mechanism for vasorelaxation [103, 118]. Conversely, levels of vasoconstrictor endothelin-1 were increased after in vitro [119, 120] and in vivo [121, 122] radiation exposure with doses ranging from 0.2 to 20 Gy. In addition, the endothelial production and release of vasoconstrictor angiotensin II by endothelial cells, in bovine pulmonary arterial endothelial cells and HUVECs [123, 124] and in pulmonary endothelial cells collected from irradiated rats [125, 126] increased dose- and time-dependently starting 24 h after exposure to 5–30 Gy. Overall, one can conclude that endothelial irradiation induces initial vasodilation during the first couple of days after irradiation, followed by chronic vasoconstriction with compromised endothelium-dependent vasodilation.

Besides affecting the endothelial layer of blood vessels, ionizing radiation can also directly affect vascular smooth muscle cells (VSMCs; Fig. 2). In culture in the absence of endothelial cells, VSMCs underwent decreased proliferation after a 1.25–20 Gy exposure [127–129], with a reduction of viable cells only 15 days after exposure [128, 129]. Surviving VSMCs demonstrated reduced contractibility [129], but maintained a contractile phenotype after exposure to 10–20 Gy [130]. In contrast, when VSMCs were co-cultured with endothelial cells and both were irradiated together with 2–10 Gy, VSMCs changed from a normal contractile to a fibrogenic phenotype [73] associated with the pathogenesis of atherosclerosis [131]. Fibrosis was induced by TGFβ released by irradiated endothelial cells, resulting in small mothers against decapentaplegic (SMAD) signaling in VSMCs [73]. Exposure to 6 Gy also mediated increased myofilament Ca^2+^ sensitivity in isolated rat thoracic aortic VSMCs 9 and 30 days after exposure [132, 133]. Furthermore, oxidative stress has been shown to induce vasoconstriction by promoting Ca^2+^ release from VSMC intracellular stores [134] and by upregulating VSMC proliferation by either their secretion of cyclophilin A [135] or by the binding of oxidative stress products hydroperoxyoctadecadienoic acids and 4-hydroxy-2-nonenal to VSMCs [136, 137]. An overview of findings supporting deterioration of vascular tone by different radiation qualities and doses is given in Table 3.

**Table 3:** Experimental findings to support the deterioration of the vascular tone by ionizing radiation

Time factor	Experimental model	Radiation quality (dose rate)	Total dose (Gray)	Experimental findings	Methods	References
Acute	Rabbit carotid artery	X-rays (3.9–4.1 Gy/min)	8, 16	Impaired acetylcholine-induced vasorelaxation 20 h after irradiation	Isometric pressure myography	[95]
Acute	HUVEC	X-rays (2.7 Gy/min)	4	Elevated protein expression of iNOS and nitrotyrosine 6 h after irradiation	Western blotting for iNOS and nitrotyrosine	[95]
Acute	BAEC/HUVEC	X-rays (0.86 Gy/min)	6	Activated eNOS signaling 12–48 h after irradiation	Western blotting for eNOS and phospo-Ser1177-eNOS	[98]
Acute	BAEC	X-rays (0.86 Gy/min)	6, 8, 10, 12, 15, 20	Elevated protein expression of eNOS 24 h after irradiation; Impaired acetylcholine-induced vasorelaxation 24 h after irradiation	Western blotting for eNOS; pressure myography	[99]
Acute	BAEC	X-rays (2.55 Gy/min)	5, 10, 15	ATM involvement in the activation of eNOS signaling 1-12 h after irradiation	Western blotting for eNOS and phospo-Ser1177-eNOS; NOS-activity assays; immunocytochemistry for ATM-pSer1981	[100]
Acute	Rabbit central ear artery	γ-rays (Co-60)	45	Impaired acetylcholine-induced vasorelaxation 1, 4, 6 and 10 weeks after irradiation	Isometric pressure myography	[101]
Acute	Rabbit central ear artery	γ-rays (Co-60)	45	Impaired acetylcholine, substance P and calcitonin gene-related peptide-induced vasorelaxation 1, 4 and 6 weeks after irradiation	Isometric pressure myography	[102]
Acute	Rabbit thoracic aorta	γ-rays (Co-60, 0.307 Gy/min)	2, 4, 6	Impaired NO-mediated acetylcholine-induced vasorelaxation 9 and 30 days after irradiation	Isometric force myography	[103]
Acute	Rat abdominal aorta	γ-rays (Co-60, 0.0875 Gy/min)	15	Impaired acetylcholine-induced vasorelaxation 18 h, 72 h and 6 months after irradiation	Isometric force myography	[104]
Fractionated	Human cervical artery	X-rays	47.9 ± 2.8	Impaired NO-mediated acetylcholine-induced vasorelaxation 4–6 weeks after radiotherapy	Electrophysiological experiments; Immunohistochemistry for eNOS	[105]
Fractionated	Human axillary artery	X-rays	not specified	Impaired endothelium-dependent vasodilation	Vascular ultrasonography	[106]
Acute	HUVEC	γ-rays (Cs-137, 1 Gy/min)	2, 4, 6, 8, 10, 12, 16, 20	Reduced IL-2 and arachidonic acid-induced cyclooxygenase activity 24 and 48 h after irradiation	Radio-immunoassay for 6-ketoprostaglandin F1α prostaglandin and thromboxane	[107]
Acute	BAEC	X-rays (0.62 Gy/min)	0.01–2	Reduced arachidonic acid-induced prostacyclin production 30 min after irradiation	Radio-immunoassay for 6-ketoprostaglandin F1α	[108]
Acute	BPAEC	γ-rays (Co-60, 1.1 Gy/min)	6, 15, 30	Elevated prostacyclin production and elevated amino-isobutyric acid uptake 24 h after irradiation	Radio-immunoassay for 6-ketoprostaglandin F1α; liquid scintillation spectrometry for [^3^H]arachidonic acid release	[109]
Acute	BAEC	γ-rays (Co-60)	0.5, 5	Elevated prostacyclin production 4 and 8 h after 5 Gy and 24 h after 0.5 Gy	Radio-immunoassay for 6-ketoprostaglandin F1α	[110]
Acute	BAEC	X-rays (1 Gy/min) γ-rays (Co-60, 5 Gy/min)	4, 5, 8 10, 12, 50	Elevated prostacyclin production, elevated arachidonic acid release and activation of cyclooxygenase 24 h after irradiation	Radio-immunoassay for 6-ketoprostaglandin F1α and thromboxane B2	[111]
Acute	BPAEC	γ-rays (Cs-137, 1.29 Gy/min)	4, 10, 20	Elevated prostacyclin production 6 h and 1, 2, 7, 14 and 21 days after irradiation	Radio-immunoassay for 6-ketoprostaglandin F1α; liquid scintillation spectrometry for [^3^H]arachidonic acid release	[112]
Acute	Rabbit abdominal aorta	γ-rays (Co-60)	10, 20, 30, 40, 50	Decreased prostacyclin production 6 h and 1–14 days after irradiation	Platelet aggregation inhibition bioassay	[113]
Acute	Human umbilical cord rings	X-rays	2	Decreased prostacyclin production 30 min after irradiation	Thin-layer radiochromatography	[114]
Acute	Rabbit abdominal aorta	γ-rays (Co-60)	1,0, 20, 30, 40, 50	Decreased prostacyclin production 1–4 months after irradiation	Platelet aggregation inhibition bioassay	[115]
Fractionated (2 or 4 sessions)	BAEC	X-rays (1 Gy/min)	4, 8	Recovery of reduced prostacyclin production 12–15 days after irradiation	Radio-immunoassay for 6-ketoprostaglandin F1α	[117]
Acute	BAEC	X-rays (1 Gy/min)	3, 6	Recovery of reduced prostacyclin production 2–10 days after irradiation	Radio-immunoassay for 6-ketoprostaglandin F1α	[117]
Acute	Rat thoracic aorta	γ-rays (Co-60, 0.8 Gy/min)	6	Impaired NO-mediated acetylcholine-induced vasorelaxation, but not endothelial hyperpolarizing factor-dependent vasorelaxation 30 days after irradiation	Isometric force myography	[118]
Acute	HUVEC	X-rays (0.2 Gy/min)	0.1	Elevated endothelin and protein expression 2 and 4 h after irradiation	RT-qPCR; Immunofluorescence for endothelin 1	[119]
Acute	BPAEC	X-rays (10 Gy/min)	5, 10, 20, 30	Increased angiotensin converting enzyme activity 24, 48 and 96 h after irradiation	Liquid scintillation counting of radioactive angiotensin converting enzyme–substrate	[123]
Fractionated (14 sessions)	EA.hy926	X-rays (2 Gy/min)	28	Elevated angiotensin II gene expression 1–5 months after last irradiation	RT-qPCR	[124]
Acute	BPAEC	γ-rays (Co-60, 2.5 Gy/min)	10, 20, 30	Angiotensin converting enzyme and plasminogen activator activity decreased linearly, and prostacyclin and thromboxane production increased linearly with increasing radiation dose	Radio-immunoassay for 6-ketoprostaglandin F1α and thromboxane B2; Fibrin plate lysis assay for plasminogen activator activity; Spectrophotometric assay for angiotensin converting enzyme activity	[125]
Acute	HMVEC–VSMC co-culture	γ-rays (Cs-137, 1 Gy/min)	2, 10	Induction of fibrogenic phenotype in vascular smooth muscle cells 24 h after irradiation	RT-qPCR for fibrogenic phenotype-related genes	[73]

*Co* Cobalt, *Cs* Cesium, *RT*-*qPCR* reverse transcriptase real-time quantitative polymerase chain reaction

## Procoagulatory and prothrombotic phenotype

**Fig. 3:**
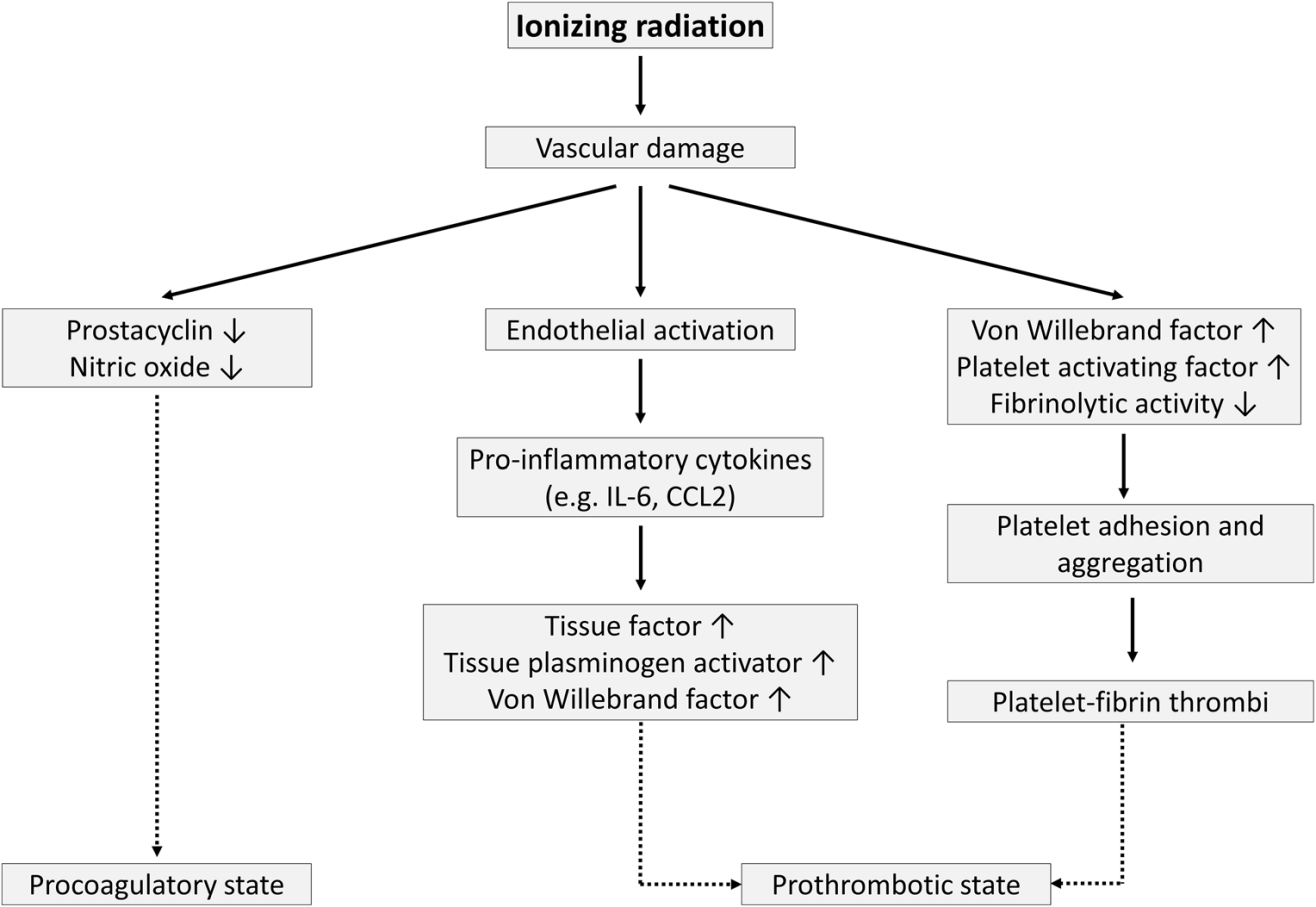
Irradiation-induced procoagulatory and prothrombotic state in endothelial cells. Endothelial irradiation results in a decreased production of anticoagulants prostacyclin and NO, resulting in a procoagulatory state. In addition, endothelial cell activation and general vascular damage result in elevated secretion of prothrombotic proteins (e.g., vWF) and a reduced fibrinolytic activity producing a prothrombotic state

In addition to altered vascular tone, vascular damage shifts the homeostatic balance towards a procoagulant and prothrombotic endothelial cell phenotype [138]. Because prostacyclin and NO are the main anticoagulatory agents secreted by endothelial cells [139], their decreased production after radiation exposure results in platelet aggregation and blood clot formation (Fig. 3). However, molecular mechanisms responsible for loss of endothelial thromboresistance are more complex. An irradiated endothelium indeed increases the synthesis of von Willebrand factor (vWF) [140–144] and platelet-activating factor [145] while reducing thrombomodulin [68, 146, 147] and prostacyclin production [108, 117, 148], as well as its fibrinolytic activity [149–151]. These changes promote platelet adhesion and aggregation and the development of platelet–fibrin thrombi [152–155]. Cytokines produced during endothelial activation (e.g., IL-6 and CCL2) further affect hemostasis by inducing the expression of tissue factor, tissue plasminogen activator, and vWF [156–158]. In this context, irradiation with 14 Gy was shown to induce atherosclerotic plaques with an inflammatory phenotype prone to hemorrhage in ApoE^−/−^ obese mice [75], which may accelerate atherosclerosis [159]. An overview of findings supporting the procoagulatory and prothrombotic effect on endothelial cells by different radiation qualities and doses is given in Table 4.

**Table 4:** Experimental findings to support the induction of a procoagulatory and prothrombotic phenotype in endothelial cells by ionizing radiation

Time factor	Experimental model	Radiation quality (dose rate)	Total dose (Gray)	Experimental findings	Methods	References
Acute/fractionated (5, 10 or 20 sessions)	HUVEC	γ-rays (Cs-137, 1 Gy/min)	20	Elevated vWF release 66 h after both acute and fractionated irradiation	ELISA for vWF	[140]
Acute	Rat heart	X-rays (1.95 Gy/min)	15, 20	Increased deposition of vWF 3 and 6 months after irradiation with, respectively, 20 and 15 Gy	Immunohistochemistry for vWF	[141]
Fractionation (1, 4, 10 or 20 sessions)	Mouse kidney	X-rays (2.35 Gy/min)	10, 12, 14, 16, 18, 20, 22, 24, 26, 32, 40	Elevated levels of glomerular vWF staining 40 weeks after irradiation	Immunohistochemistry for vWF	[142]
Acute	HUVEC	γ-rays (Cs-137, 5.77 Gy/min)	20, 30, 40	Elevated secreted vWF 24, 48 and 72 h after irradiation	Immunoprecipitation and gel electrophoresis of vWF	[143]
Acute	BAEC	X-rays (2.4 Gy/min)	20	Elevated secreted and intracellularly stored vWF 48 h after irradiation	ELISA for vWF	[144]
Fractionation (8 or 16 sessions)	Rat intestine	X-rays (2.01 Gy/min)	33.6, 67.2	Reduced thrombomodulin immunoreactivity in all types of vessels 2 weeks after irradiation	Immunohistochemistry for thrombomodulin	[146]
Acute	HUVEC	γ-rays (Co-60, 1.21 Gy/min)	6.25, 12.5, 25, 50	Elevated thrombomodulin release and activity 6 and 24 h after irradiation followed by a decline in thrombomodulin release and activity 2, 4 and 6 days after irradiation	Radio-immunoassay for soluble thrombomodulin; Immunocytochemistry for thrombomodulin	[147]
Fractionated (23 sessions)	Dog liver	γ-rays (Co-60)	46	Decreased vascular fibrinolytic activity 24 and 30 months after irradiation	Fibrin slide technique	[149]
Acute	BAEC	X-rays (0.62 Gy/min)	0.01–2	Reduced arachidonic acid-induced prostacyclin production 30 min after irradiation	Radio-immunoassay for 6-ketoprostaglandin F1α	[108]
Fractionated (2 or 4 sessions)	BAEC	X-rays (1 Gy/min)	4, 8	Recovery of reduced prostacyclin production 12–15 days after irradiation	Radio-immunoassay for 6-ketoprostaglandin F1α	[117]
Acute	BAEC	X-rays (1 Gy/min)	3, 6	Recovery of reduced prostacyclin production 2–10 days after irradiation	Radio-immunoassay for 6-ketoprostaglandin F1α	[117]
Fractionated	Human superficial epigastric vein	X-rays	20–43	Decreased vascular fibrinolytic activity 5-13 weeks and 2.5 years after radiation therapy	Fibrin slide technique	[150]
Acute	Rat lung	γ-rays (Co-60, 3 Gy/min)	25	Decreased fibrinolytic activity 2, 3, 4, 5 and 6 months after irradiation	Fibrin slide technique	[151]
Acute	Mouse	γ-rays (Co-60, 2.03–2.08 Gy/min)	6	Elevated platelet aggregation rate 4 h and 1, 3, 5 and 7 days after irradiation	Aggregometry	[152]
Acute	Rat	γ-rays (Co-60, 2 Gy/min)	8	Elevated platelet aggregation rate 4 h and 1, 3, 5 and 7 days after irradiation	Aggregometry	[152]
Acute	Rabbit	γ-rays (Co-60, 0.99 Gy/min)	4	Elevated platelet aggregation rate 4 h and 1, 3, 5 and 7 days after irradiation	Aggregometry	[152]
Fractionated (23 sessions)	Dog liver	γ-rays (Co-60)	46	Increased platelet aggregation and adhesiveness 2 weeks after irradiation	Photoelectric method platelet aggregation; rolling tube platelet adhesiveness test	[154]
Acute	ApoE^−/−^ mice	X-rays	8, 14	Increased thrombomodulin and tissue factor level 4 weeks after irradiation	Immunohistochemistry for thrombomodulin and tissue factor	[159]

*Co* Cobalt, *Cs* Cesium, *ELISA* enzyme-linked immunosorbent assay

## Endothelial cell retraction and death

**Fig. 4:**
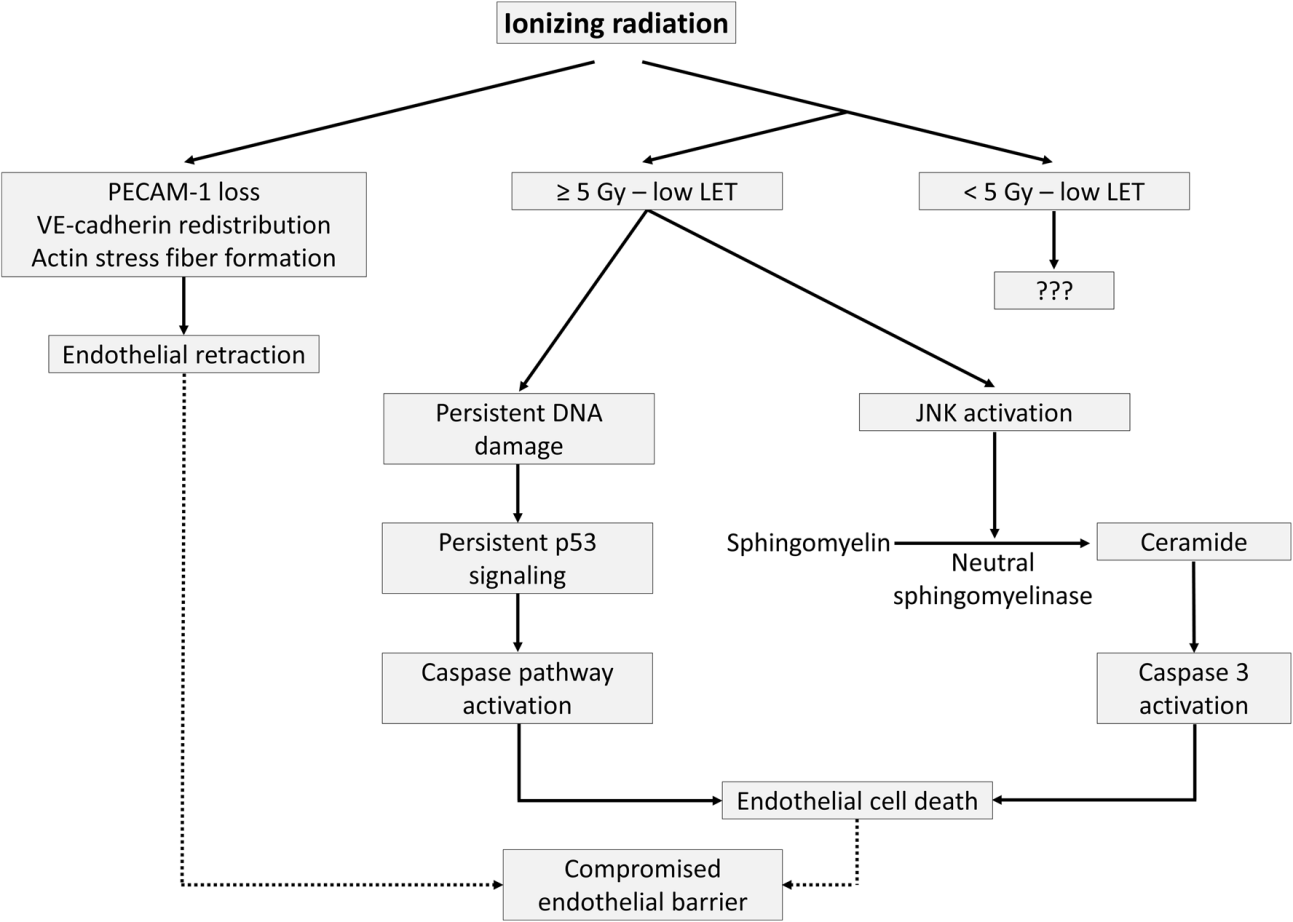
Irradiation-induced retraction and death of endothelial cells. Ionizing radiation exposure is able to decrease PECAM-1 expression, redistribute VE-cadherin, and produce actin stress fibers leading to endothelial retraction. Depending on the radiation dose, radiation quality, and inherent radiation sensitivity, ionizing radiation can activate the caspase pathway by ceramide formation and persistent p53 signaling, causing endothelial cell death. As a consequence of endothelial retraction and cell death, the physiological endothelial barrier is compromised

Besides edema formation in surrounding tissues caused by endothelial inflammation and tissue injury [160, 161], exposure to radiation doses as low as 2 Gy can induce a transient and rapid decrease in the integrity of in vitro human endothelial barriers through cell detachment and loss of platelet endothelial cell adhesion molecule (PECAM)-1 [162, 163] (Fig. 4). Rapid loss of endothelial monolayer integrity depends on cytoskeletal reorganization due to actin stress fiber formation and redistribution of vascular endothelial (VE)-cadherin junctions, resulting in endothelial retraction [164–167]. At higher doses, a more direct cause of increased vascular permeability is of course endothelial cell death [168, 169]. Sensitivity of endothelial cells to cell-reproductive death after ionizing radiation can be assessed by clonogenic assays, the method of choice in such situation [170]. Radiosensitivity varies between endothelial cells from different vascular beds, with HUVECs being the most sensitive and HHSEC being the most radioresistant among the tested ones [171]. In addition, sensitivity to cell-reproductive death depends on radiation quality, with the relative biological effectiveness of α-particles estimated at 5.5 and 4.6 for 10% survival of A549 cells and EA.hy926 cells, respectively [172]. Doses as low as 0.1 Gy can reduce the surviving fraction of EA.hy926 cells [172, 173]. Doses higher than 5 Gy induce endothelial cell apoptosis by the production of ceramide [174, 175]: irradiation activates stress-activated c-Jun N-terminal kinases (JNKs), resulting in the conversion of sphingomyelin to ceramide by neutral sphingomyelinase and the subsequent activation of caspase-3 [176, 177]. In addition, endothelial apoptosis at doses higher than 5 Gy can also be induced by persistent DNA damage, resulting in p53 accumulation and activation of the caspase pathway [178, 179]. Mechanisms behind endothelial cytotoxicity of lower doses are less known. For example, apoptotic EA.hy926 cell death was not increased after exposure to 0.2 Gy, but well after exposure to 5 Gy [180]. In another study, TNF-α-activated endothelial cells were shown to have a discontinuous induction of apoptosis, with a relative maximum at 0.3 Gy and 3 Gy and a relative minimum at 0.5 Gy [82]. In addition, our group observed a dose-dependent increase in endothelial cell apoptosis from 0.5 Gy in HUVECs and from 0.1 Gy in EA.hy926 cells [173]. In vivo, compromised barrier function is involved in the pathogenesis of vascular failure, including atherosclerosis [23, 181, 182]. An overview of findings supporting the induction of endothelial cell retraction and cell death by different radiation qualities and doses is given in Table 5.

**Table 5:** Experimental findings to support the induction of endothelial cell retraction and death by ionizing radiation

Time factor	Experimental model	Radiation quality (dose rate)	Total dose (Gray)	Experimental findings	Methods	References
Acute	HBMVEC/HUVEC	γ-rays (Cs-137, 0.85 Gy/min)	5	Decreased transendothelial resistance 2–4 h after irradiation, Elevated number of holes in monolayer 3 h after irradiation, uncoupling of PECAM-1 3 h after irradiation	Transendothelial resistance measurement; permeability assay; immunocytochemistry for PECAM-1	[162]
Acute	HUVEC	X-rays (0.8–0.9 Gy/min) γ-rays (Cs-137, 0.85 Gy/min) Protons (0.1–1 Gy/min) Helium (0.1–1 Gy/min)	5 5 2 5	Reduced transendothelial resistance, uncoupling of PECAM-1 and release of endothelial microparticles 3 h after irradiation	Transendothelial resistance measurement; immunocytochemistry for PECAM-1	[163]
Acute	HMVEC	X-rays (0.5 Gy/min)	5, 10, 20	Enhanced actin remodeling, reduced endothelial adherens junctional integrity and elevated endothelial monolayer permeability 6–24 h after irradiation	Immunofluorescence for F-actin and VE-cadherin; permeability assay	[164]
Acute	BPAEC	γ-rays (Cs-137)	2, 5, 10	Elevated permeability 2, 2.5 and 3 h after irradiation	Microcarrier bead based permeability assay	[165]
Acute	MPMEC	X-rays (2.05 Gy/min)	0.125, 0.25, 0.50, 1	Increased endothelial cell death 2 days after 2.5 Gy irradiation; Time and dose-dependent cellular retraction and F-actin depolarization 1–24 h after irradiation	Clonogenic assay, bright field image analysis; immunocytochemistry for F-actin	[166]
Acute	MPMEC	X-rays (2.75 Gy/min)	0.5, 1, 2	Endothelial cell retraction 4–8 h after irradiation, linked to acute edema formation	Phase contrast photo microscopy, immunocytochemistry for F-actin, lung wet weight determination	[167]
Acute	HUVEC/HHSEC/HBMVEC/HOMVEC/HPMEC/HDMEC	γ-rays (Cs-137, 2–3 Gy/min)	2, 4, 6	Difference in radiation sensitivity between different endothelial cells	Clonogenic assay	[171]
Acute	EA.hy926	X-rays (0.855 Gy/min) Alpha (1 Gy/min)	2, 4, 6, 8, 10 0.5, 1, 1.5, 2	Difference in radiation sensitivity towards different radiation types	Clonogenic assay	[172]
Acute	HUVEC	X-rays (0.25 Gy/min)	0.5, 5	Elevated apoptosis 24, 48 and 72 h after irradiation	Flow cytometry with annexin-V and propidium iodide	[173]
Acute	EA.hy926	X-rays (0.25 Gy/min)	0.1, 0.5, 5	Elevated apoptosis 24, 48 and 72 h after irradiation	Flow cytometry with annexin-V and propidium iodide	[173]
Acute	BAEC	γ-rays (Cs-137, 1 Gy/min)	10	Elevated ceramide levels 30 s to 10 min after irradiation	Diacylglycerol kinase assay	[174]
Acute	BAMVEC	X-rays (1.24 Gy/min)	2–10	Elevated endothelial apoptosis 6 h after irradiation	DNA fragmentation assay	[179]
Acute	EA.hy926	γ-rays (Co-60, 0.02 or 0.19 Gy/min)	5	Elevated endothelial apoptosis 24 hafter irradiation	Flow cytometry with caspase-3 activity assay and SubG1 fraction analysis	[180]
Acute	EA.hy926	X-rays (4 Gy/min)	0.3, 0.7, 1, 3	Elevated endothelial apoptosis 24 hafter irradiation	Flow cytometry with annexin-V and propidium iodide; Caspase 3/7 activity assay	[82]

*Co* Cobalt, *Cs* Cesium

## Mitochondrial dysfunction

Recent years have seen increasing interest for radiation-induced mitochondrial dysfunction as a cause of endothelial dysfunction in the context of cardiovascular disease [183–187]. In most mammalian cells, mitochondria are primarily considered as the major suppliers of cellular energy in the form of ATP produced by oxidative phosphorylation (OXPHOS) [186]. However, mitochondria are only present in modest number in endothelial cells [185, 188] and produce a low proportion of the total amount of cellular energy [189–191]. Thus, endothelial mitochondria are more likely to primarily serve as important signaling organelles [192]. While mitochondria are linked to endothelial function (reviewed in [185]) and endothelial mitochondria are known to play a role in vascular diseases (reviewed in [186]), data on the effect of ionizing radiation on endothelial mitochondria in general are scarce. It was shown that in vitro endothelial cells exposed to 5–20 Gy of γ-rays lose their mitochondrial membrane potential and that mitochondrial ROS production increased 24–72 h after exposure [193]. Furthermore, murine cardiac microvascular endothelial cells irradiated with 8 and 16 Gy X-rays acquired protein expression profiles associated with mitochondrial dysfunction [194]. In light of the caveats in current knowledge, sections below will focus on three main mitochondrial functions hypothesized to be disturbed in endothelial cells after exposure to ionizing radiation: Ca^2+^ regulation, control of cell death, and oxidative stress signaling.

Normal cytosolic Ca^2+^ concentrations are maintained approximately 10,000 times lower than extracellular Ca^2+^ concentrations by plasma membrane and endoplasmic reticulum Ca^2+^ ATPases. Because these transport proteins require ATP for Ca^2+^ transport, mitochondria are indirectly involved in this form of Ca^2+^ regulation [195, 196]. In addition, mitochondria can also directly sequester Ca^2+^ and, thereby, regulate intracellular concentrations by their inner membrane uniporter rapid mode of Ca^2+^ uptake into heart mitochondria (RaM), which is driven by the proton electrochemical potential. Conversely, mitochondria release Ca^2+^ via the 2Na^+^/Ca^2+^- and 2H^+^/Ca^2+^-exchanger. Increased mitochondrial Ca^2+^ activates dehydrogenase enzymes in mitochondria and increases ATP synthase activity, leading to increased NADH and ATP production [197]. Sparse evidence exists that altered mitochondrial calcium contributes to endothelial dysfunction in cardiovascular diseases. For example, in diabetes, high glucose levels were shown to elevate mitochondrial Ca^2+^ levels in human endothelial cells, thereby increasing mitochondrial free radical production [198]. Furthermore, mitochondrial Ca^2+^ regulates the intensity of TNF-α-induced inflammation in mouse lung microvascular endothelium [199]. In addition, flow-induced dilation of human coronary arterioles was found to be mediated by Ca^2+^ influx via the transient receptor potential vanilloid type 4 (TRPV4) channel that is in closely apposition with endothelial mitochondria, resulting in mitochondrial ROS release in coronary artery endothelial cells [200]. Finally, the mitochondrial Ca^2+^ uniporter can potentiate endothelial cell migration [201], and its levels are markedly decreased in endothelial cells derived from CVD patients [202]. While it is known that mitochondrial Ca^2+^ signaling is affected by ionizing radiation (reviewed in [203, 204]), there is a lack of experimental studies on the role of mitochondrial Ca^2+^ in the irradiated endothelium.

Importantly, mitochondria are also central executioners of apoptosis. In normal state, anti-apoptotic proteins of the B-cell lymphoma (Bcl)-2 family located on the outer mitochondrial membrane inhibit pro-apoptotic effector proteins Bcl-2-associated protein X (BAX) and Bcl-2 homologous antagonist killer (BAK) [205]. In response to cytotoxic stress, Bcl-2 homology 3 (BH3)-only proteins inhibit Bcl-2 proteins, resulting in BAX and BAK activation. BAX and BAK form oligomers that permeabilize the mitochondrial outer membrane, mediating the release of cytochrome c into the cytosol [206, 207]. Cytosolic cytochrome *c* promotes the activation of caspase 9 by apoptotic protease activating factor 1 (APAF1), which in turn activates effector caspases that induce cell death [208]. Dysregulation of these vital functions can promote endothelial inflammation, apoptosis, and senescence, which are all linked to the development and progression of atherosclerosis [169, 185–187, 209].

As a byproduct of OXPHOS, a small amount of O_2_ undergoes monoelectronic reduction mainly at complexes I and III of the mitochondrial electron transport chain, resulting in the generation of O_2_^·−^ [210]. As a result, mitochondria are a main generation site of ROS within cells [210]. Formed O_2_^·−^ is converted to H_2_O_2_ by SOD2 inside mitochondria, which is able to activate the redox-sensitive transcription factors NRF2, AP-1, and NF-κB [44]. Because mitochondria are an important cellular source of ROS [169, 211, 212], they are closely related to oxidative stress signaling. At relatively low levels, mitochondria-derived ROS are signaling molecules that support normal or compensatory cellular functions involved in hypoxic adaptation, immunity, cellular differentiation, and longevity [213]. However, excessive mitochondrial ROS levels can cause oxidative stress [186]. This is problematic, because mitochondria themselves are critical targets of ROS [214], leading to irreversible damage to mitochondrial DNA, membrane lipids, and proteins [215].

In an effort to combat oxidative challenge, ROS and RNS activate cellular transcription factors, NRF2, NF-κB, and AP-1, resulting in increased expression of ROS-detoxifying enzymes catalase, SOD, glutathione peroxidase (GPx), glutathione S-transferases (GST), and heme oxygenase-1 (HO-1) [216–218]. NRF2 is believed to be the main regulator of cellular resistance to pro-oxidants. Because NRF2 controls basal and induced expression of an array of antioxidant response element-dependent genes, including *HO*-*1*, *SOD2,* and *GPx* [219, 220], it is not surprising that this factor is induced after radiation exposure in both normal and cancerous cells (0.05–8 Gy) [217, 221, 222]. NRF2 also confers cellular radioresistance [223–226] by mediation of DNA repair, by regulating genes from the homologous recombination DNA repair pathway, and oxidative defense in both normal and cancerous cells [222, 227]. In addition, NRF2 upregulation has been implicated in oxidative stress-induced endothelial dysfunction [228]. Because NRF2 mediates gene expression resulting in both high NADPH production and the production, regeneration and utilization of GSH, thioredoxin, and peroxiredoxin, upregulation of NRF2 leads to increased levels of these antioxidants after irradiation (0.25–20 Gy) in lymphocytes and glioma cells [229, 230]. Of note, elevated levels of several mammalian peroxiredoxin isoforms have been evidenced after a 10 Gy radiation exposure of mouse testis and liver, further enhancing cellular defense mechanisms [231–234]. Both cumulative and acute radiation exposure can disrupt the cellular redox balance. However, oxidative stress only prevails when pro-oxidant levels eventually overwhelm cellular antioxidant systems, an event marked by enzyme inactivation, a low GSH/glutathione disulfide ratio, and a decreased pool of low molecular weight antioxidants. The consequence of such redox imbalance is manifested by modifications of nucleic acids, lipids, proteins, and other biomolecules [216, 235].

If radiation doses are high enough to overwhelm cellular antioxidant responses, oxidative stress can induce mitochondrial dysfunction by ROS-induced ROS release [236]. As a consequence, radiation-induced oxidative stress that normally disappears within seconds after exposure [214] can lead to the initiation of a self-amplifying cycle, giving rise to long-term ROS production [237] and concomitant mitochondrial dysfunction [169]. During this process, mitochondrial DNA seems to be particularly sensitive to oxidative damage because of its limited DNA repair capacity, lack of protective histones, a high exon to intron ratio and its close proximity to the electron transport chain [238]. In agreement, a range of studies demonstrated changes in mitochondrial function and number after exposure of cells or tissues to high doses of ionizing radiation [239–242]. Doses of 5–20 Gy of γ-radiation were found to induce a dose-dependent increase in ROS levels with a decrease in mitochondrial activity [193].

Furthermore, 15 Gy of X-rays induced persistent oxidative stress in endothelial cells, linked to mitochondrial dysfunction and premature senescence [243]. Effects of low radiation doses have been less studied on mitochondrial dysfunction in endothelial cells. Doses of 1.5 Gy, 4 Gy, and 10 Gy were found to influence mitochondrial membrane potential in HUVECs 2 days after exposure. While the mitochondrial potential reverted back to control level by days 5 and 6 in 1.5 and 4 Gy irradiated cells, respectively, 10 Gy resulted in persistently decreased mitochondrial activity [244]. In another example, the respiratory capacity of cardiac mitochondria was significantly reduced 40 weeks after local heart irradiation in ApoE^−/−^ mice with a single X-ray dose of 2 Gy [245]. In addition, 0.1 Gy and 0.5 Gy were found to reduce mitochondrial signaling in murine hippocampus and cortex [246]. An overview of findings supporting the induction of mitochondrial dysfunction in endothelial cells by different radiation qualities and doses is given in Table 6.

**Table 6:** Experimental findings to support the induction of mitochondrial dysfunction in endothelial cells by ionizing radiation

Time factor	Experimental model	Radiation quality (dose rate)	Total dose (Gray)	Experimental findings	Methods	References
Acute	HUVEC	γ-rays (5.104 Gy/min)	5, 10, 20	Elevated apoptosis levels 24, 48 and 72 h after irradiation; Elevated reactive oxygen species production, reduced mitochondrial membrane potential, inhibition mitochondrial permeability transition pore opening 24 h after irradiation	Flow cytometry with annexin-V and propidium iodide, CM-H_2_DCFDA and JC-1; spell out assay	[193]
Acute	MCMVEC	X-rays	8, 16	Increased ICAM-1 and ICAM-2 protein expression levels and enriched proteins linked to mitochondrial dysfunction pathway 16 weeks after irradiation	Flow cytometry for ICAM-1 and ICAM-2; Proteomics	[194]
Acute	HPMVEC	X-rays	15	Induced mitochondrial dysfunction with reduced complex II activity, higher mitochondrial mass and increased mitochondrial superoxide production 14, 21 and 28 days after irradiation	Flow cytometry with mitosox and mitotracker; Seahorse metabolism analysis	[243]
Acute	HUVEC	X-rays (1.5 Gy/min)	1.5, 4, 10	Reduced mitochondrial membrane potential 3–7 days after irradiation	Flow cytometry with JC-1	[244]

JC-1, 5,5′,6,6′-Tetrachloro-1,1′,3,3′-tetraethylbenzimidazolylcarbocyanine iodide

## Premature endothelial senescence

Aging of the vascular system predisposes the cardiovascular system to the development of diseases, even in the absence of other risk factors [247]. On a cellular level, vascular aging corresponds to endothelial cell senescence [248, 249], a phenomenon that refers to irreversible arrest of endothelial cell renewal. At a molecular level, senescence is induced and maintained by p53 and p16-Rb pathways that inhibit cell-cycle progression [250]. Both pathways are activated either during attrition of telomeres, referred to as replicative senescence [251, 252], or during stress situations independently of telomere shortening, referred to as stress-induced premature senescence [253]. For instance, limited availability of nutrients and growth factors, chromatin perturbations, improper cell contacts, and oxidative stress prematurely induces senescence via cell stress [254]. Oxidative stress is of special importance, because it induces and accelerates senescence at multiple molecular levels: accelerated telomere shortening [254], induction of DNA damage leading to p53 activation [255], NO scavenging decreasing its bioavailability [256], and mitochondrial dysfunction [257].

Endothelial senescence is currently emerging as a contributor to the pathogenesis of atherosclerosis by increasing ROS production, decreasing NO availability, and increasing the production of pro-inflammatory molecules IL-6, IL-1, IL-8, CCL2, and ICAM-1 [257–259]. All these molecules indeed contribute to the development and progression of atherosclerosis [209, 257], as indirectly evidenced by the presence of senescent endothelial cells in human atherosclerotic plaques [258]. These observations have to be taken with caution: identification of senescent endothelial cells suggests an association with atherosclerosis, but cannot be used to evidence a causal relationship between endothelial senescence and development and/or progression of atherosclerosis. Considering that ionizing radiation induces oxidative stress [254, 260], DSBs [261], and telomere shortening [260], it is not surprising that it constitutes a stressor that can evoke premature senescence in cells. Several in vitro studies demonstrated that high dose (4–50 Gy) [45, 262–267] and medium dose (0.5 Gy) [79] radiation exposure as well as chronic radiation exposure to low doses [268–270] induces premature endothelial cell senescence. An overview of findings supporting the induction of premature senescence in endothelial cells by different radiation qualities and doses is given in Table 7.

**Table 7:** Experimental findings to support the induction of premature senescence in endothelial cells by ionizing radiation

Time factor	Experimental model	Radiation quality (dose rate)	Total dose (Gray)	Experimental findings	Methods	References
Acute	HPMVEC	X-rays	1, 5, 15	Increased senescence and associated secretory phenotype 14, 21 and 28 days after irradiation	Senescence associated β-galactosidase staining, Immunocytochemistry for p16Ink4a, p21Waf1 and γH2AX expression, ELISA for IL-8 secretion and DNA damage response activation	[243]
Acute	TICAE	X-rays	10	Elevated senescence 5 days after irradiation	Senescence associated β-galactosidase staining	[262]
Acute	HUVEC	γ-rays (Cs-137, 2 Gy/min)	2, 4, 8	Elevated senescence 6, 8, 11, 14, 17 and 21 days after irradiation	Senescence associated β-galactosidase staining	[45]
Acute	BAEC	γ-rays (Co-60, 0.62 Gy/min)	8	Elevated senescence 5 days after irradiation	Senescence associated β-galactosidase staining	[263]
Acute	BAEC/HUVEC	γ-rays (Co-60, 0.72 Gy/min)	4, 8	Elevated senescence 5 days after irradiation	Senescence associated β-galactosidase staining	[264]
Acute	HUVEC	γ-rays (Cs-137, 2.82 Gy/min)	2, 4	Elevated senescence 2 days after irradiation	Senescence associated β-galactosidase staining	[265]
Acute	BPAEC	X-rays (2.4 Gy/min)	50	Elevated senescence 24, 72 and 120 h after irradiation	Senescence associated β-galactosidase staining	[266]
Acute	BAEC	γ-rays (Cs-137, 5 Gy/min)	5, 10, 15	Elevated senescence 3 and 6 weeks after irradiation	Senescence associated β-galactosidase staining, digital image analysis	[267]
Chronic	HUVEC	γ-rays (Cs-137, 0.0041 Gy/min)	2.066, 4.133	Elevated senescence 3 and 6 weeks after irradiation, insulin-like growth factor binding protein 5 signaling	Senescence associated β-galactosidase staining; microarray analysis	[268]
Chronic	HUVEC	γ-rays (Cs-137, 0.0041 Gy/min)	2.066, 4.133	Elevated senescence and reduced of PI3 K/Akt/mTOR Pathway 10 and 12 weeks after irradiation	Senescence associated β-galactosidase staining; proteomics	[269]
Chronic	HUVEC	γ-rays (Cs-137, 0.0024 Gy/min)	4.032	Elevated senescence 3 and 6 weeks after irradiation, enrichment of senescence-related biological pathways	Senescence associated β-galactosidase staining; proteomics; western blot for PI3 K, mTOR, Akt	[270]
Acute	TICAE	X-rays (0.5 Gy/min)	0.5, 2	Elevated senescence 14 days after irradiation	Senescence associated β-galactosidase activity assay, multiplex bead array for IGFBP7	[18]

## Conclusions

**Fig. 5:**
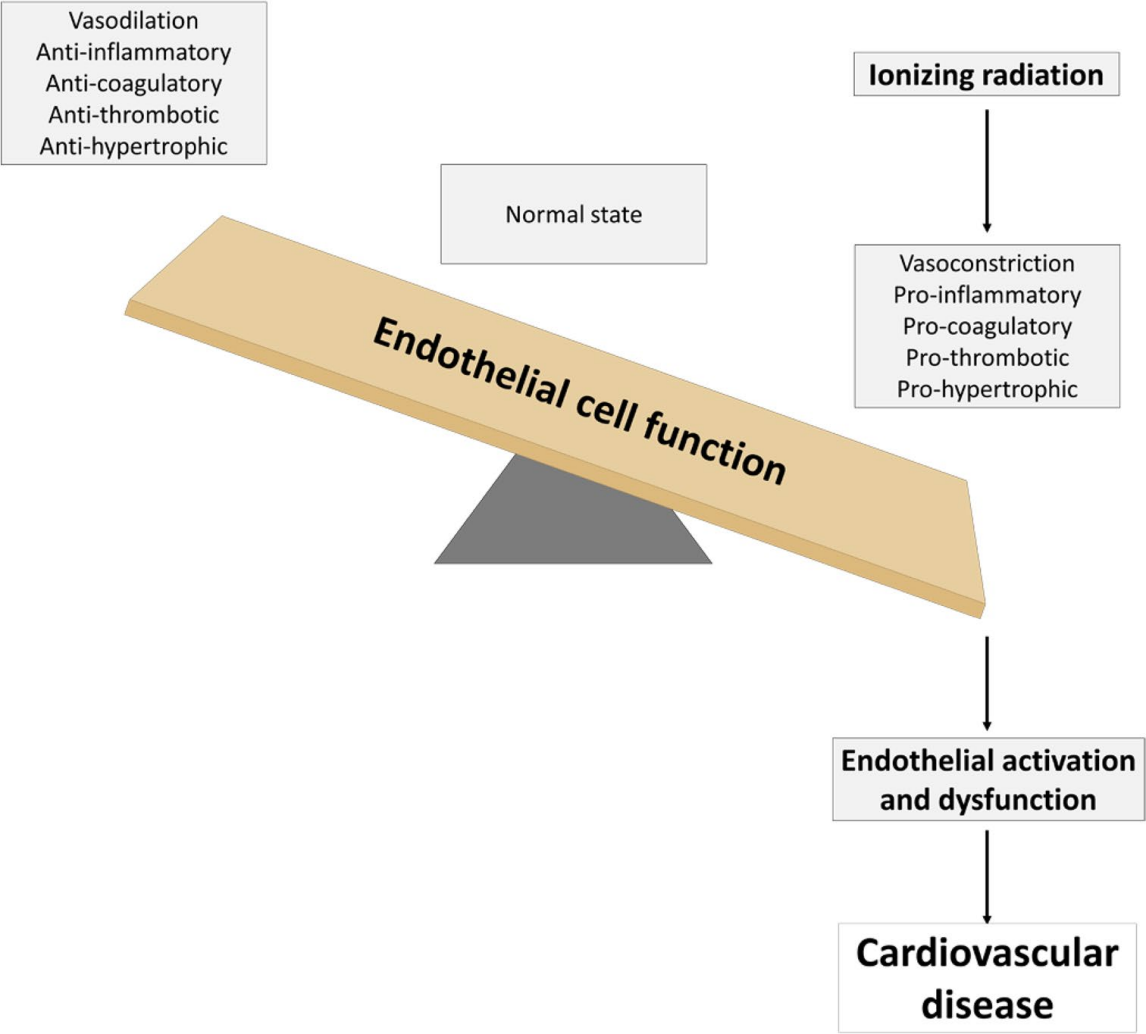
Ionizing radiation can induce both endothelial cell activation and dysfunction. The resulting vasoconstrictive, pro-inflammatory, procoagulatory, prothrombotic, and prohypertrophic environment can initiate and/or trigger the progression of several pathological cardiovascular conditions, together with other vascular risk factors (e.g., dyslipidemia and hypertension)

In current times, it has become a common practice to use ionizing radiation during justified diagnostic and therapeutic medical procedures. High doses of ionizing radiation lead to cardiac dysfunction over time [29, 30]. However, precise biological and molecular mechanisms are still elusive. In this review, we substantiated the hypothesis that ionizing radiation exposure can induce endothelial activation and dysfunction (Fig. 5). Mechanisms of action predominantly involve induction of a pro-inflammatory state by genotoxic stress, oxidative stress and DAMP release, deterioration of the vascular tone by compromising endothelium-dependent vasodilatation, induction of a procoagulatory and prothrombotic endothelial phenotype by perturbing hemostasis, inducing endothelial cell retraction and death leading to loss of vessel integrity, and induction of mitochondrial dysfunction and premature senescence. Together with other vascular risk factors (e.g., dyslipidemia and hypertension), ionizing radiation can initiate and/or trigger the progression of several pathological conditions, such as atherosclerosis and subsequent radiation-induced heart disease. While these effects are well documented at high doses, there is to date little knowledge on the effect at low doses of ionizing radiation. Furthermore, little is known about the effects of chronic radiation exposure, the exposure to different kinds of radiation qualities (in particular particle radiation), the evolution of endothelial dysfunction in time, and the role of endothelial heterogeneity. Future studies are required to advance the understanding of the mechanisms that lead to endothelial dysfunction, as they may relate to the induction and/or progression of cardiovascular diseases following exposure to ionizing radiation. Further understanding of radiation-induced endothelial dysfunction could lead to advances in the development of countermeasures, such as antioxidant therapy [271, 272], for cardiovascular disease in radiation-exposed individuals.
